# Applying Machine learning to analyze the utilization of insecticide-treated nets among rural under-five children in East Africa

**DOI:** 10.1016/j.parepi.2026.e00511

**Published:** 2026-05-12

**Authors:** Fentahun Bikale Kebede, Amanuel Worku, Angwach Abrham Asnake, Eliyas Addisu Taye, Kussie Urmale Mare, Abiy Mergia Bulto, Abraham Keffale Mengistu, Tsegereda Abebe Andargie, Bewuketu Terefe

**Affiliations:** a Strategic Affairs Executive Office, Ministry of Health, Addis Ababa, Ethiopia; b Addis Ababa University, Addis Ababa, Ethiopia; c Department of Epidemiology and Biostatistics, School of Public Health, College of Health Science and Medicine, Wolaita Sodo University, Wolaita Sodo, Ethiopia; d Department of Health Informatics, Institute of Public Health, College of Medicine and Health Science, University of Gondar, Gondar, Ethiopia; e Department of Nursing, College of Medicine and Health Sciences, Semera University, Semera, Ethiopia; f Planning, reporting, and Accountability Division, Africa Centers for Disease Control and Prevention, Addis Ababa, Ethiopia; g Department of Health Informatics, College of Medicine Health Science, Debre Markos University, Debre Markos, Ethiopia; h International Institute for Primary Health-Care, Addis Ababa, Ethiopia; i School of Nursing, College of Medicine and Health Science, University of Gondar, Gondar, Ethiopia

**Keywords:** Insecticide-treated nets, Under-five children, Machine learning, Rural East Africa

## Abstract

**Introduction:**

Malaria continues to be a major cause of morbidity and mortality in children under the age of five in the sub-Saharan region of Africa. Despite being one of the pillars of prevention, the use of Insecticide-Treated Nets (ITNs) is still low in rural East Africa. This research aimed to forecast the use of ITNs by rural communities with children in this region using machine learning (ML) and identify the most predictive factors.

**Methods:**

We analyzed pooled data from the Demographic and Health Surveys (DHS) in 11 East African nations between 2011 and 2022. Six ML classification models, K-Nearest Neighbours, Support Vector Classifier (SVC), Random Forest, Multilayer Perceptron (MLP), Naïve Bayes, and Logistic Regression, were developed to predict ITN adoption. Model performance was evaluated using Accuracy, Precision, Recall, F1-Score, and ROC-AUC. Shapley Additive exPlanations (SHAP) analysis was used to improve model interpretability and to rank feature importance.

**Result:**

In 98,684 weighted household samples, the prevalence of general ITN use was 47.12%. The MLP model generalized best overall, achieving the highest accuracy (91.56%) and ROC-AUC (97.15%), with very high precision (87.95%) and recall (95.24%). While the SVC model had better recall (99.92%), its precision (84.19%) was significantly lower, indicating a higher proportion of false positives. MLP's F1-score of 91.39% restored it as the most balanced classifier in this scenario. SHAP analysis identified the number of children who slept under ITNs and the number of ITNs in the home as the strongest predictors. More community-level wealth, household wealth, and media exposure were also positively associated with ITN use.

**Conclusion:**

Machine learning made accurate predictions of ITN use, with the MLP model having the best and most balanced performance. SHAP allowed us to uncover actionable reasons for the most influential drivers of ITN use. These evidence-based outcomes can inform targeted public health interventions to improve ITN coverage and utilization in high-risk rural settings, ultimately reducing malaria cases and deaths.

## 1 Introduction

Based on the 2022 World Health Organization (WHO) report, nearly 3.3 billion people across 97 countries remain at risk of contracting malaria, with the highest burden shouldered by sub-Saharan Africa (SSA) (Organization WH, 2022). Pregnant women and children under the age of five are disproportionately affected, accounting for the majority of malaria morbidity and mortality (Organization WH, 2022; diseases NIoAai, 2011; Dombrowski et al., 2018). Furthermore, based on the African health overview, 41% of the projected 9.7 million under-five child deaths worldwide occur in SSA (Altındiş and Izulla, 2010). SSA countries continue to bear a significant share of the global burden of under-five mortality, despite the impressive progress made toward achieving the SDGs target in reducing the child mortality rate to 25 per 1000 live births in all nations by 2030 (Unicef, 2017; General A, 2015).

Malaria is an infectious disease with a significant impact on global morbidity and mortality in millions of people (Alonso and Tanner, 2013; Tanser and Le Sueur, 2002). In low and middle-income countries, malaria is the fourth most common cause of death in children under five. Globally, as reported in 2018, an estimated 220 million cases and 405,000 deaths are caused by malaria (Aborode et al., 2021; Mbanefo and Kumar, 2020). Among these, 93% of cases and 94% of deaths were malaria-related and occurred in Africa (Zhao et al., 2020; Nghochuzie et al., 2020). In Africa alone, approximately 285,000 children under five years of age died (Report WM, 2019). Malaria affects the most vulnerable age group, which is children under five years old. Approximately 67% (272,000) of all malaria-related deaths occurred globally in 2018 (Organization WH, 2019). The prevalence of malaria in children under five years of age in SSA ranges from 0.7% to 80.3% (Sarfo et al., 2023). Malaria is the top cause of death among children under five years, particularly in SSA, and it is preventable by the use of ITN, indoor insecticide chemical spraying, prompt and effective treatment of malaria cases, and access to preventive measures for pregnant women and children (Aheto et al., 2023).

To combat malaria in youngsters, careful consideration of close follow-up and continuous research is important. The use of ITNs among rural children under five years of age in East Africa is crucial for reducing the burden of malaria. Malaria constitutes a significant risk to children under five years of age due to high morbidity and mortality rates. ITN utilization has been evidenced to be an efficient and effective measure in controlling malaria. However, its low utilization (Yeboah et al., 2023), communities from rural areas with poor access to health services, as well as other sociodemographic characteristics, are more likely to have morbidity and mortality among children under the age of five (Seyoum et al., 2023; Yaya et al., 2019). Although malaria remains the primary cause of morbidity and mortality in SSA, particularly in East Africa, reports reveal that ITNs are underutilized (Organization WH, 2017a; Organization WH, 2017b) and that there is a paucity of data on the factors influencing ITN use among children under 5 years of age. Compared to metropolitan areas, fatality and improper use of ITN are more common in rural areas (Seyoum et al., 2023; Yaya et al., 2019; Organization WH, 2017a). In sub-Saharan Africa (SSA), household use of ITNs is influenced by multiple factors, including the availability of nets, the number of children under five in the household, and overall family size. In larger households, caregivers often prioritize children under five and pregnant women for ITN use, which may limit access for other members (Azanaw and Worede, 2025; Konlan et al., 2022a; Tassew et al., 2017). Similarly, in East Africa, the low utilization of ITNs among rural children under five is linked to several interrelated factors. Limited awareness and understanding of ITNs, inadequate supply, competing household demands due to multiple young children, and poor knowledge contribute to low adoption rates. In addition, cost and accessibility barriers, complaints of skin irritation, as well as cultural, social, and geographical factors, are more pronounced among rural populations (Yeboah et al., 2023; Seyoum et al., 2023; Kebede et al., 2023; Konlan et al., 2022b; Solanke et al., 2023).

Numerous previous studies (Lencha and Deressa, 2015; Biadgilign et al., 2012; Gobena et al., 2012; Kyalo and Kioko, 2018) have primarily focused on identifying determinants and associated factors of ITN utilization among children under five using conventional statistical approaches. While these methods have been valuable in highlighting key associations, they are generally based on predefined assumptions, such as linear relationships and restricted interaction structures, which may limit their ability to fully capture the complexity of real-world data (Bizzego et al., 2021; Ij, 2018). This limitation becomes particularly important when analyzing large-scale, nationally representative datasets such as the Demographic and Health Surveys (DHS), which are characterized by high-dimensional variables, potential nonlinear relationships, and complex interactions across multiple levels. Although some findings from prior studies as noted in the discussion have already identified important predictors of ITN utilization, they have largely relied on traditional modeling frameworks. The novelty of the present study lies in moving beyond these conventional approaches by applying machine learning techniques to re-examine ITN utilization patterns within the same epidemiological context. Unlike earlier studies, this approach enables the identification of nonlinear relationships, higher-order interactions, and complex patterns that may remain undetected using standard regression-based analyses. Furthermore, this study also focused on disadvantaged group of rural under five children in East Africa where significant equalities are observed between urban and rural residencies.

Machine learning algorithms are designed to learn directly from data and optimize predictive performance without imposing strict parametric assumptions (Sarker, 2021). As a result, they are particularly effective in uncovering hidden structures, complex dependencies, and nonlinear associations within large and heterogeneous datasets (Dhar, 2013; Beam and Kohane, 2018). Despite these advantages, the application of machine learning to understand and predict ITN utilization—especially among rural households with under-five children in East Africa—remains limited.

To address this gap, this study applies multiple machine learning models to predict ITN utilization and identify its key determinants using nationally representative DHS data from East Africa. Beyond simply identifying associated factors, this study contributes to the literature by (i) improving predictive accuracy, (ii) capturing complex and previously unobserved relationships among determinants, and (iii) providing a comparative methodological perspective between traditional and machine learning approaches. These contributions offer more nuanced and data-driven insights that can inform targeted malaria prevention strategies and strengthen evidence-based policy design, thereby advancing both the methodological and applied dimensions of existing research.

## 2 Methods

### 2.1 Study design, settings, and period

Various data sources can be used to produce data pertinent to health planning, program implementation, monitoring, and evaluation in developing nations. The most significant source of data is the routine health information system, which is based on health facility data and population-based surveys. (Boerma and Sommerfelt, 1993). We used data from the National Demographic and Health Survey (DHS) of East Africa. The DHS Program provides technical assistance through the World Bank, the United Nations Children's Fund (UNICEF), the United States Agency for International Development (USAID), and ICF to implement population and health surveys in nations worldwide. The DHS was conducted in a population-based cross-sectional study every five years in developing countries. This study used recent DHS data from East African nations. Data was collected between 2011 and 2022. As presented below (Table 1), only 11 of the 17 countries in East Africa provided complete DHS data regarding the use of ITNs.

**Table 1:** Countries, sample size, and survey year of demographic and health surveys included in the analysis for 11 East African countries.

Country	Survey year	Weighted sample size	Weighted frequency
Burundi	2016/17	11,863	12.02
Kenya	2022	11,824	11.98
Comoros	2012	2464	2.50
Madagascar	2021	10,811	10.96
Malawi	2016/17	15,399	15.60
Mozambique	2011	7842	7.95
Tanzania	2022	7510	7.61
Rwanda	2016	6841	6.93
Uganda	2016	12,770	12.94
Zambia	2018	6618	6.71
Zimbabwe	2015	4742	4.80

#### 2.1.1 Sampling methods and population

The DHS used a stratified two-stage sampling method for each nation. In the first step, Enumeration Areas (EAs) representing the entire nation were randomly drawn from the sample frame (based on the most recent national census available). Within each administrative geographic region, DHS samples are usually separated into urban and rural areas. The second stage was systematic sampling of homes within each cluster or EA. The target population (men and women aged 15 to 64 years) was interviewed in a few selected households at this stage. Following the extraction of the pooled data, the sample size was determined by the availability of the outcome variable in each DHS and comprised all families with children under the age of five across the 11 East African nations. Finally, this study included a total weighted sample of 98,684 children from 11 countries.

#### 2.1.2 Data sources

Secondary data analysis was performed using the DHS dataset. Only children under 5 years old living in rural areas of East African countries were included, based on surveys conducted between 2011 and 2022.

#### 2.1.3 Variables of study

ITN usage among children under five was the study's outcome variable. If a child under the age of five slept in an ITN the night before each survey, the answer was classified as “YES” =1, and if not, it was classified as “NO” =0. The outcome variable was determined following the guidelines outlined in the DHS Statistics Handbook (Croft et al., 2020). The independent variables of this study were age of the household head (<24, 25–34, and > 35 years old), sex of the household head (male, female), household wealth index poorest, poorer, middle, and rich), family size (one up to five, and greater than five), number of under five children (one, two, and more than two), household educational attainment (not educated/educated), household owned radio, and television (yes/no), number of under five children slept under ITN (not at all, all of them, some of them), number of ITNs in the household (0, 1, 2, 3 and more than 3), number of rooms used for sleeping(1, 2,3, and more than three), household has mosquito bed net for sleeping (yes, no), birth order (first, second or third, and more than third), community wealth level (low, high), community level media exposure status (low, high), and community level education (low, high), were included. Independent variables were classified based on a review of the literature.

#### 2.1.4 Operational definitions for community-level variables

Because the community-level components could not be observed or recorded as individual data during the survey, all components were estimated from aggregate values derived from the individual records. Each was estimated based on the value of each distinct variable, even if the methodology was the same as in other studies. In this study, a cluster or primary sample unit in the dataset shared by a group of families was referred to as a community-level factor. Combining components at the individual, group, and community levels to produce variables. Community household head's education, community media exposure, and community wealth were among the community variables. Community-level variables, which are continuous, were further categorized into high and low using mean/median values based on their distributions to make the results easier to grasp (Nkoka et al., 2019; Terefe et al., 2023; Ntenda and Chuang, 2018).

**Community household heads' education**: defined as the proportion of household heads in each cluster with at least a secondary education. The variable was categorized as ‘low’ or ‘high’ based on the median (56%), with clusters having proportions less than or equal to the median classified as low and those above the median as high.

**Community household head media exposure:** was derived by aggregating individual household head responses on exposure to at least one form of media (radio, television, or newspapers) within each cluster. The proportion of household heads exposed to media was calculated for each cluster and categorized as ‘low’ and ‘high’ levels. Clusters with less than 60% of household heads exposed to media were classified as having low media exposure, while those with 60% or more were classified as having high media exposure.

**Community household head wealth:** The same process was used to derive this variable from each household's wealth index. In a community's two lowest wealth quintiles, it was considered high if between 55% and 100% of women in the household, and low if between 0% and 54%.

#### 2.1.5 Data quality, collection tools, and procedures

In the DHS, a pre-test was conducted before data collection, a debriefing session with pre-test fieldworkers was held, and changes to the questionnaires were made as necessary. DHS guidance provides more details about the data-collection process (Croft et al., 2018). Every five years, the DHS collects data by trained professional data collectors who are nationally representative and reflect the demographic and health challenges unique to each nation using five different surveys. These included questionnaires for households, women, men, biomarkers, and health institutions. The person record questionnaire used in this study was used to ascertain regional ITN use and its contributing factors (Croft et al., 2020; Croft et al., 2018).

#### 2.1.6 Data management processes and analysis

Python programming software version 3.12 was used throughout the data analysis in this study. Machine learning models were implemented using the scikit-learn library (version 1.4.2), with supporting packages such as pandas (version 2.2.1) for data manipulation and NumPy (version 1.26.4) for numerical operations. Data preprocessing involves several activities, such as data integration, data cleaning to remove noise, handling missing data and outliers, and transformation, depending on the nature of the data. The missing values were handled using mode imputation techniques for categorical variables. Non-relevant features were removed from the dataset.

#### 2.1.7 Data cleaning and integration

**Fig. 1:**
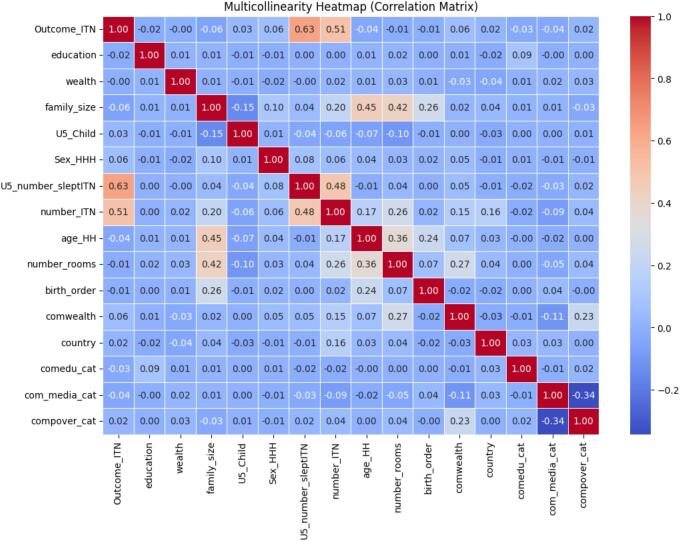
Correlation matrix.

The data used in this study were collected from various DHS records and merged into a single structured format. Irrelevant features were identified and removed to improve the dataset's quality. Inconsistencies, duplicate variables, and formatting issues in the dataset were verified and corrected to maintain consistency across variables. Missing values were analyzed for prevalence and distribution throughout the dataset. For categorical variables, missing values were imputed using the mode if fewer than 25% of values were missing, consistent with standard recommendations for handling low-to-moderate missingness in categorical data (García-Laencina et al., 2010). This was selected to preserve the categorical nature of the data while avoiding information loss. Variables were transformed using appropriate techniques: one-hot encoding was applied to nominal features (Sex of household head, household education attainment, ownership of radio and television, Household has ITN for sleeping, Community wealth level, community level media exposure status, community level education status). Label (ordinal) encoding was applied to ordered categorical variables (age of the household head, household wealth index, family size, number of under-five children, number of under-five children slept under ITN, number of ITNs in the household, number of rooms used for sleeping, and birth order). To explore the relationship between variables, a correlation matrix (Fig. 1) was computed. This allowed us to identify highly correlated variables that would introduce redundancy to the model. Multicollinearity was also tested with the Variance Inflation Factor (VIF). Variables with high collinearity were explored to ensure that predictors in the final models were independent and uniquely added to the analysis.

#### 2.1.8 Dataset partitioning

The study used an 80/20 train-test split and 10-fold cross-validation to ensure robust model evaluation and performance assessment. The dataset was divided into training and testing sets, with the former used to build and train the model and the latter used to evaluate its performance on previously unseen data. Furthermore, 10-fold cross-validation was used to validate the model's performance by dividing the data into 10 subsets. The model was trained and tested 10 times, with each test set as a separate fold and the remaining folds used for training. This method reduces overfitting and yields a more reliable estimate of the model's generalization performance (Brownlee, 2018).

#### 2.1.9 Hyperparameter tuning and model development

In addition to logistic regression, six machine-learning classifiers were employed to predict ITN utilization among children under five. These classifiers included K-Nearest Neighbours (KNN), Random Forest (RF), Support Vector Classifier (SVC), Multilayer Perceptron (MLP), Naïve Bayes (NB), and Logistic Regression (LR). The performance of each model was evaluated using five key evaluation metrics: Accuracy, Precision, Recall, F1-Score, and ROC-AUC.

To optimize each model's performance, a GridSearchCV strategy was employed, specifying a set of hyperparameter values for each model based on model-specific requirements and previous research. To find the best configuration, a grid search systematically evaluated all possible combinations of these parameters: number of neighbours and distance metrics for KNN; number of trees, maximum depth, and minimum samples per split for RF; the regularization parameter, kernel type, and gamma for SVC; the number of hidden layers, neurons, and learning rate for MLP; and regularization strength and penalty type for LR.

Following hyperparameter optimization, the tuned models were evaluated on the test dataset. Since the outcome variable is categorical with two mutually exclusive groups, the problem was approached as a binary classification task. To identify the best-performing model, we compared classifiers using key evaluation metrics, including accuracy, precision, recall, F1-score, and ROC-AUC curve (Kuhn and Johnson, 2013). Cross-validation was conducted to assess the consistency of each model's performance across different data subsets. The final model was selected based on its ability to balance predictive accuracy and generalization to unseen data, thereby ensuring its reliability in accurately predicting ITN use.

#### 2.1.10 Model explanation and feature importance

The Shapley additive Explanations (SHAP) approach was designed to show the relationship between the outcome variables and the predictors (Konlan et al., 2022a), which evaluates the contribution of each feature to the model's prediction, offering both global and local interpretability through a game-theoretic framework (Bifarin, 2023). Feature selection was performed to identify the most influential factors affecting healthcare access predictions. SHAP was chosen for its ability to provide clear, interpretable insights into how each feature impacts model decisions, an essential aspect in healthcare applications where transparency and explainability are critical (Bifarin, 2023; Lundberg and Lee, 2017). Feature selection was performed to identify the most influential features for predicting ITN utilization. SHAP was chosen because it provides clear, interpretable insights into how each feature contributes to the model's decisions, which is crucial in healthcare applications where interpretability is (Nohara et al., 2021).

#### 2.1.11 Ethical considerations and data access

ICF International and respective country-level Institutional Review Boards obtained ethical approval for the primary DHS data collection. Permission to access and use the datasets for this study was granted by the DHS Program (www.dhsprogram.com). As this research involved the secondary analysis of existing, de-identified, publicly available data, no additional Institutional Review Board (IRB) approval was sought. All data were treated with strict confidentiality. The DHS program rigorously maintained informed consent, confidentiality, anonymity, and participant privacy during the original data collection.

## 3 Results

### 3.1 Sociodemographic characteristics of the study participants

This survey comprised a weighted sample of 98,684 households with children under five. Regarding household head age, approximately 56,957 (57.72%) participants were over 35 years old. Similarly, 74,440(73.13%) participants were male household heads. In terms of the educational level of the household head, 98,307 (99.62%) participants were not enrolled in formal schooling. Regarding the household wealth index, approximately 28,702 (29.08%) households were found in the poorest wealth quantile; however, more than half, 52,185 (52.88%), and 42,980 (43.55%) of the households had more than five family members and two children, respectively. Of these, 27,729 (28.10%) had no ITN. Regarding community-level characteristics, 66,488 (67.37%) and 50,309 (50.98%) households had high community-level education and mass media exposure, respectively (Table 2).

**Table 2:** Socio-demographic-related characteristics of respondents on ITN utilization among rural under-five children in East African countries.

Variables	Count	Frequency
Age of the household head		
<24	7898	8.00
25–34	33,829	34.28
>35	56,957	57.72

Sex of the household head		
Male	74,440	75.43
Female	24,244	24.57

Wealth index		
Poorest	28,702	29.08
Poorer	25,672	26.01
Middle	22,404	22.70
Rich	21,906	22.20

Family size		
≤5	46,499	47.12
>5	52,185	52.88

Number of children under five		
One	37,619	38.12
Two	42,980	43.55
More than two	18,086	18.33

Education		
Not educated	98,307	99.62
Primary and higher	377	0.38

The number of children Under five slept under the ITN		
No	43,364	44.13
Yes, all of them	44,882	45.67
Yes, some of them	10,027	10.20

Number of ITNs		
0	27,729	28.10
1	24,413	24.74
2	23,576	23.89
3	13,171	13.35
>3	9795	9.93

Number of rooms used for sleeping		
1	31,745	32.17
2	38,224	38.73
3	20,461	20.73
>3	8255	8.36

Has a mosquito bed net for sleeping		
No	27,729	28.10
Yes	70,955	71.90

Birth order		
First	12,440	12.61
2nd or 3rd	20,284	20.55
More than third	65,961	66.84

Community-level education		
Low	32,196	32.63
High	66,488	67.37

Community-level media exposure		
Low	48,375	49.02
High	50,309	50.98

Community wealth level		
High	49,495	50.16
Low	49,189	49.84

### 3.2 Prevalence of ITN utilization among children under five in East Africa

**Fig. 2:**
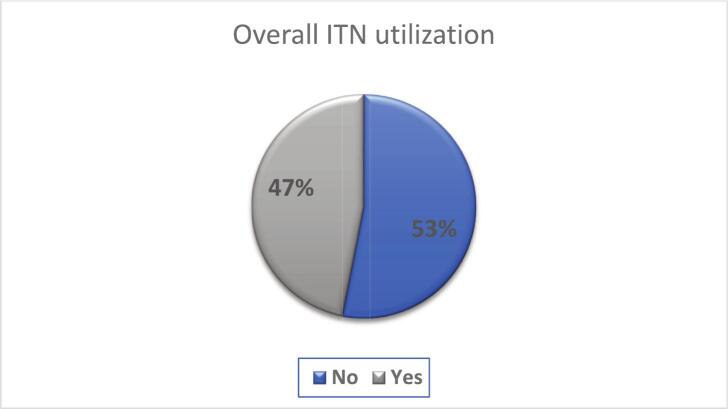
Overall ITN Utilization in East African rural under-five children from 2011 to 2022.

**Fig. 3:**
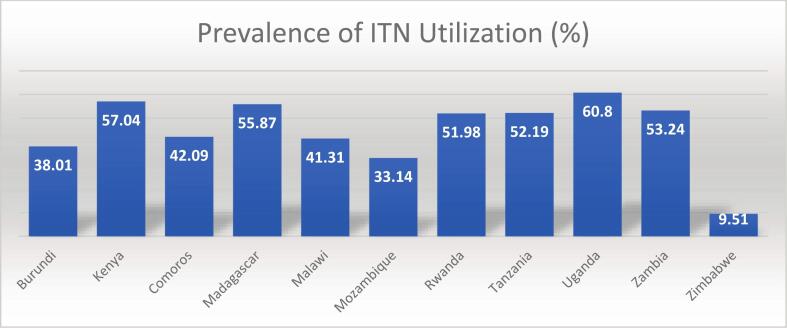
Prevalence of ITN utilization across countries among children under five in East Africa from 2011 to 2022.

The overall prevalence of ITN use among under-five children in East Africa was 47.12% (95% CI 46.81, 47.43) (Fig. 2). The highest prevalence was observed in Uganda (60.80%), and the lowest was in Zimbabwe (9.51%). Burundi, Kenya, Madagascar, and Mozambique scored less than 50% (Fig. 3).

### 3.3 Machine learning analysis

All six models (Table 3) were highly accurate in predicting ITN utilization, with MLP (91.56%) and SVC (91.07%) being the most accurate. Their discriminative abilities, however, were Random Forest (96.35%) and MLP (97.15%) according to their highest ROC-AUC values, indicating that they could differentiate more effectively between ITN users and non-users. Out of all the models employed, KNN was moderately good, with an accuracy was 88.57%. While it had excellent recall (93.03%), i.e., it could accurately classify most ITN users, its accuracy (84.44%) was not particularly high, suggesting some misclassification of non-users. Logistic regression also achieved 87.86% accuracy with excellent recall (93.45%), but comparatively lower ROC-AUC (88.42%), suggesting that while helpful, it may not capture all the nuances in the data as well as non-linear models.

**Table 3:** performance comparison of machine learning models for predicting ITN use in Rural East Africa.

Model	Accuracy (%)	Precision (%)	Recall (%)	F1-Score (%)	ROC-AUC (%)
KNN	88.57	84.44	93.03	88.53	94.83
Logistic Regression	87.86	83.07	93.45	87.95	88.42
Random Forest	89.71	87.41	91.47	89.39	96.35
Support Vector Classifier (SVC)	91.07	84.19	99.92	91.38	96.19
Multi-Layer Perceptron (MLP)	91.56	87.95	95.24	91.45	97.15
Naïve Bayes	91.54	87.33	96.11	91.51	95.12

**Fig. 4:**
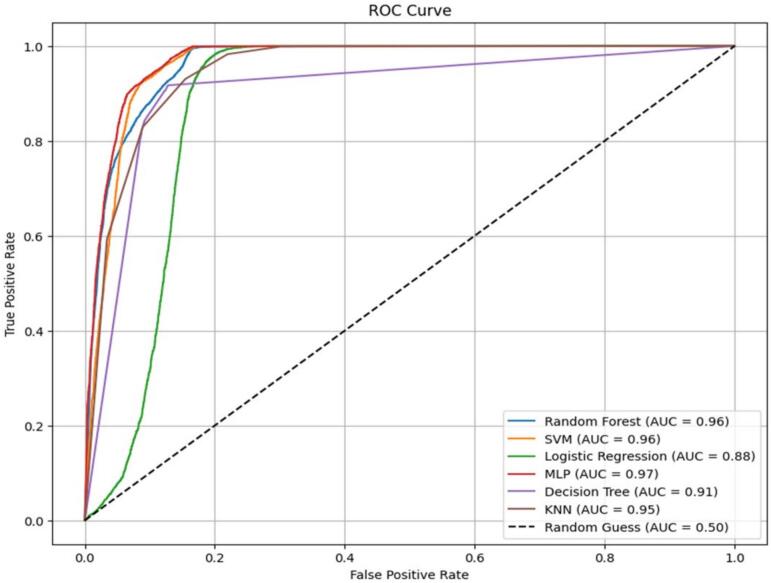
AUC of machine learning models for predicting ITN use in Rural East Africa.

Random Forest, with 89.71% accuracy, was also one of the most well-balanced models, scoring high precision (87.41%) and recall (91.47%), although with a lower ROC-AUC (96.35%). Nevertheless, it was a good classifier for ITN usage. SVC also achieved 91.07% accuracy and an extremely high recall of 99.92%, i.e., correctly classifying nearly all ITN users. However, its accuracy (84.19%) was also slightly lower, with a higher rate of false positives. The MLP model was the most accurate, with the best accuracy (91.56%), ROC-AUC (97.15%), precision (87.95%), and recall (95.24%), and therefore the most balanced and effective classifier in this study. Naïve Bayes also performed nearly as well as MLP, with 91.54% accuracy, high recall (96.11%), and a good ROC-AUC (95.12%), making it another strong contender despite its independence assumption (Fig. 4).

### 3.4 SHAP important for predictor identification

**Fig. 5:**
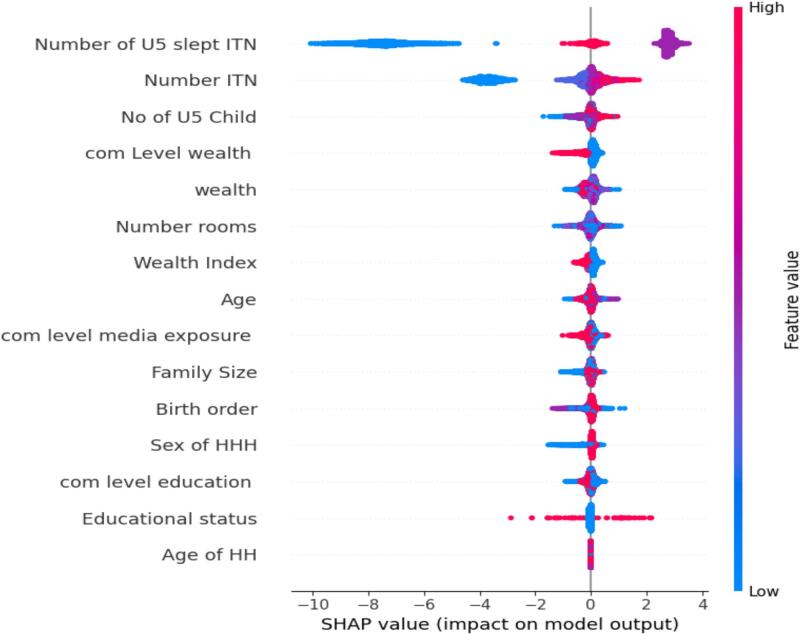
Feature importance.

The SHAP summary plot (Fig. 5) presents the most important features influencing the prediction of bed net use among children under five. The number of children who slept under ITNs and the total number of ITNs in the household were the most influential predictors. Interestingly, households with a low number of under-five children and a low number of ITNs were associated with a higher predicted likelihood of bed net use, likely reflecting a pattern where available nets were prioritized for the few children present. In addition, higher community-level wealth, greater household wealth, and greater community-level media exposure were positively associated with bed net utilization, highlighting the role of socioeconomic and informational factors. A low family size also contributed positively, possibly indicating that smaller households were better able to allocate nets per child. Other contributing variables included the number of rooms, birth order, educational status, and the age of the household head, though their effects were relatively small. Overall, the plot reveals that both individual- and community-level socioeconomic conditions, as well as household composition, significantly influence bed net use among children.

## 4 Discussion

The current study showed that machine learning (ML) algorithms can effectively classify and predict ITN utilization by children in East Africa. These models identified significant predictors of ITN use with high precision and provided valid predictions for implementing targeted malaria prevention strategies. A total of six ML algorithms were employed, including logistic regression, k-nearest neighbours (KNN), random forest (RF), support vector classifier (SVC), multilayer perceptron (MLP), and naïve Bayes (NB). These models were trained on a comprehensive dataset including sociodemographic, household, community, and behavioral factors. This strategy supports strong prediction of ITN use and underscores the use of ML to guide evidence-based interventions and optimize resource allocation in malaria-endemic areas. Ultimately, the study aimed to improve child health outcomes through more targeted and effective delivery of ITN distribution initiatives across East Africa.

The Multilayer Perceptron (MLP) was selected as the most effective model for predicting ITN utilization, demonstrating a robust balance of precision (87.95%), recall (95.24%), and ROC-AUC (97.15%). While the Support Vector Classifier (SVC) achieved a near-perfect recall (99.92%), its substantially lower precision (84.19%) indicated a high rate of false positives, misclassifying non-users as users. In the context of public health resource allocation, such a model would be suboptimal, as it would misdirect interventions to households predicted to be non-compliant that are actually already using ITNs. The MLP's superior balance, captured by its higher F1-score, makes it more reliable for efficiently targeting resources toward true non-user households. This performance also underscores the ability of non-linear classifiers, such as MLPs, to capture the complex relationships inherent in socio-demographic data, a finding supported by prior health-related studies (Madububambachu et al., 2024; Kalipe et al., 2018; Oguntimilehin et al., 2024). However, the model's applicability is likely specific to the features and region of this dataset, and its performance should be validated on external data before broader deployment (Oguntimilehin et al., 2024).

The SHAP summary plot showed that the most important features influence the prediction of bed net use among children under five. The number of children who slept under ITNs and the total number of ITNs in the household were the most influential predictors, followed by the household's number of under-five children.

Households with a low number of under-five children and a low number of ITNs were associated with a higher predicted likelihood of bed net use, likely reflecting a pattern in which available nets were prioritized for the few children present. A study in sub-Saharan Africa has found that household ITN use is not only influenced by availability but also by the number of users for those resources (Koenker et al., 2023). A multi-country analysis reveals that when ITN-to-person ratios are low, caregivers tend to prioritize young children or pregnant women for ITN use, particularly in households with fewer children under five years of age (Eisele et al., 2011). Another study also supported this evidence, showing that the number of under-five children sleeping under an ITN was significantly higher in smaller family sizes and among those with fewer under-five children, even after adjusting for ITN ownership (Doe et al., 2024). These findings suggest that the adequacy, not just the ownership, of ITNs plays a key role in usage patterns.

### 4.1 Strengths and limitations of the study

This study offers several important strengths. First, it employed advanced machine learning techniques, using six different classification models to assess insecticide-treated net (ITN) use among children under five. This approach provided strong comparative insights into model performance. Notably, the Multilayer Perceptron model showed high accuracy and generalizability, underscoring the promise of deep learning in public health monitoring. Second, the analysis was based on a large, representative dataset of over 98,000 weighted observations from 11 East African countries, enhancing the relevance and applicability of the findings across diverse rural settings. Third, the use of SHAP analysis added an interpretable layer to the results by identifying the most influential factors driving ITN use, thereby improving the models' transparency and practical value. Finally, the study's focus on rural populations addresses a significant research gap, as these communities carry the heaviest malaria burden and are often overlooked in technology-driven health research.

However, several limitations should be noted. The study relied on cross-sectional Demographic and Health Survey (DHS) data, which prevents causal conclusions about the relationships between predictors and ITN use. Additionally, much of the data was self-reported, which may introduce recall or social desirability bias, particularly around sensitive health behaviors. The models were constrained by the variables available in the DHS, leaving out potentially relevant factors such as seasonal trends, recent ITN distribution efforts, or household sleeping arrangements. The absence of longitudinal data also limits the ability to examine changes over time or assess the lasting effects of interventions. These limitations should be carefully considered when interpreting the results or designing future studies and policies. A further limitation is that the 80/20 train-test split was not stratified by country or outcome. Given the significant regional heterogeneity, this may have introduced sampling bias, potentially affecting the generalizability of the model predictions across all East African nations.

## 5 Conclusion

This study successfully applied multiple machine learning algorithms to predict ITN usage among children in rural East Africa. Among all tested models, the Multilayer Perceptron performed best with high accuracy and robust predictive power. Key determinants of ITN utilization included the number of ITNs in the household, the number of under-five children sleeping under ITNs, household wealth, community education, and media exposure, underscoring the interplay between household and community-level factors in ITN adoption.

### 5.1 Recommendations

Based on the findings of this study, it is recommended that malaria prevention programs in East Africa focus on targeted distribution strategies to households with larger family sizes and multiple under-five children, as these were key predictors of ITN utilization. Community-based education initiatives should be intensified, particularly in areas with low media exposure and low educational attainment, to raise awareness about the benefits and correct use of ITNs. Public health campaigns should consider the behavioral and contextual factors uncovered by machine learning models, particularly those emphasized through SHAP analysis, to design more customized and effective interventions. Additionally, health ministries and NGOs are encouraged to adopt machine learning models as part of their regular planning and evaluation of malaria control programs. These tools can help inform real-time decisions and improve the efficient allocation of resources to areas with the greatest need.

## Authors' contribution

FBK and BT were involved in the conceptualization, design, data extraction, analysis, and writing of the manuscript. AW and EAT analysis, language editing, and original manuscript writing. KUM, AAA, AMB, and AKM reviewed the study design, draft manuscript, and analysis, making significant contributions. The authors have approved the final version of the manuscript.

## CRediT authorship contribution statement

**Fentahun Bikale Kebede:** Writing – review & editing, Writing – original draft, Visualization, Validation, Supervision, Software, Resources, Methodology, Investigation, Funding acquisition, Formal analysis, Data curation, Conceptualization. **Amanuel Worku:** Software, Resources, Funding acquisition, Formal analysis, Data curation. **Angwach Abrham Asnake:** Writing – original draft, Supervision, Software. **Eliyas Addisu Taye:** Visualization, Formal analysis, Data curation. **Kussie Urmale Mare:** Writing – review & editing. **Abiy Mergia Bulto:** Formal analysis, Data curation, Conceptualization. **Abraham Keffale Mengistu:** Writing – review & editing. **Tsegereda Abebe Andargie:** Software, Resources, Funding acquisition, Formal analysis, Data curation. **Bewuketu Terefe:** Writing – original draft, Visualization, Supervision.

## Consent for publication

Not applicable.

## Funding

No funding.

## Declaration of competing interest

The authors declare that they have no known competing financial interests or personal relationships that could have appeared to influence the work reported in this paper.

## Data Availability

All data concerning this study are presented in this document. The detailed dataset is freely accessible on the www.dhsprogram.com website.
